# Multi-Omics Reveal the Improvements of Nutrient Digestion, Absorption, and Metabolism and Intestinal Function via GABA Supplementation in Weanling Piglets

**DOI:** 10.3390/ani14223177

**Published:** 2024-11-06

**Authors:** Yan Zeng, Hong Hu, Yiwen He, Zhiying Deng, Yiting Guo, Xihong Zhou

**Affiliations:** 1 Key Laboratory of Agro-Ecological Processes in Subtropical Region, Institute of Subtropical Agriculture, Chinese Academy of Sciences, Changsha 410125, China; zengyan23@mails.ucas.ac.cn; 2 College of Advanced Agricultural Sciences, University of Chinese Academy of Sciences, Beijing 101408, China; 3 Key Laboratory of Livestock and Poultry Resources (Pig) Evaluation and Utilization, Ministry of Agriculture and Rural Affairs, Beijing 100125, China; huhong7777777@126.com (H.H.); 17674632896@163.com (Z.D.); guoyiting729@gmail.com (Y.G.); 4 College of Animal Science and Technology, Hunan Agricultural University, Changsha 410128, China; 5 Hunan Provincial Key Laboratory of Animal Intestinal Function and Regulation, College of Life Sciences, Hunan Normal University, Changsha 410081, China; heyiwen@hunnu.edu.cn

**Keywords:** gut microbiota, inflammation, nutrient metabolism, weanling piglets, γ-aminobutyric acid

## Abstract

The nonprotein amino acid γ-aminobutyric acid (GABA) can enhance intestinal function in piglets; however, the mechanisms involved are not yet fully understood. To explore the effects of GABA and its underlying mechanisms, weanling piglets were randomly assigned to three groups, receiving either a basal diet or a basal diet supplemented with GABA (80 mg/kg or 120 mg/kg). The results demonstrated that dietary GABA improved growth performance and reduced diarrhea incidence (*p* < 0.05). Additionally, GABA supplementation decreased the serum and intestinal levels of pro-inflammatory cytokines (*p* < 0.05), and improved intestinal morphology. Multi-omics analyses were employed to explore the alterations caused by GABA supplementation and elucidate the related mechanisms. Microbiota profiling revealed improved beta-diversity and changes in the composition of ileal bacteria and fungi. Amino acid metabolism, lipid metabolism, and digestive processes were primarily enriched in the GABA group according to metabolomics analysis. A transcriptome analysis showed significant enrichment in ion transmembrane transport and nutrition absorption and digestion pathways in the ileum. Furthermore, increased lipase and trypsin activity, along with the elevated expression of tight junction proteins confirmed the beneficial effects of GABA on intestinal nutrient metabolism and barrier function. In conclusion, dietary 80 mg/kg GABA supplementation improved nutrient digestion and absorption and intestinal function in weanling piglets.

## Simple Summary

As a nonprotein amino acid, γ-aminobutyric acid (GABA) plays a vital role in intestinal function. This study aimed to investigate the underlying mechanisms by which GABA affects intestinal health in piglets. The results showed that GABA significantly improved growth performance, reduced diarrhea, and mitigated inflammation. Additionally, microbiota profiling revealed enhanced diversity and changes in the composition of ileal bacteria and fungi. Metabolomic and transcriptomic analyses suggested that dietary supplementation with GABA could affect several pathways related to nutrient digestion and absorption. We further confirmed that GABA increased the activities of lipase and trypsin, as well as elevated the expression of tight junction proteins. Therefore, we propose that dietary 80 mg/kg GABA supplementation may enhance nutrient digestion and absorption, thereby improving intestinal function in weanling piglets.

## 1. Introduction

Weaning represents a critical transition in the growth and development of piglets, involving not only a fundamental shift from a diet of breast milk to solid feed but also adjustments to various environmental factors such as temperature, humidity, and social interactions [1]. Additionally, weanling piglets, lacking maternal antibody protection and with an underdeveloped immune system, are particularly susceptible to infections from exogenous pathogens [2]. Their compromised intestinal digestion and absorption capabilities further exacerbate weaning stress [3]. This stress manifests in reduced feed intake, slow weight gain, impaired intestinal barrier function, increased incidence of diarrhea, and even mortality [4]. Such issues contribute to significant economic losses in the modern intensive pig industry.

Prolonged or excessive use can disrupt gut microbiota balance and contribute to antibiotic resistance [5]. Consequently, exploring non-antibiotic alternatives to effectively mitigate weaning stress and support healthy growth has become a crucial focus in animal science research. Dietary supplementation with amino acids, fatty acids, and other nutrients has been shown to benefit weanling piglets [6,7]. The nonprotein amino acid γ-aminobutyric acid (GABA) has gained attention for its diverse roles in regulating intestinal motility, carcinogenesis, pain management, and inflammation [8,9]. Recent studies demonstrate that GABA supplementation for three weeks significantly boosts the growth rate of 21-day-old weanling piglets [10]. Additionally, GABA increases concentrations of IL-1β, IFN-γ, IL-4, and IL-17 in the jejunum of ETEC-infected piglets, enhancing pathogen defense [11]. Maternal GABA supplementation improves antioxidant capacity and reduces placental oxidative stress [12]. Furthermore, GABA influences gut microbiota, promoting microbial diversity and altering dominant communities in *Enterotoxigenic Escherichia coli*-challenged piglets [13]. It also affects the relative abundances of specific low-abundance bacteria [11] and supports the intestinal epithelial barrier, as some microbiota produce GABA themselves [14,15].

These studies suggest that GABA may play a beneficial role in alleviating weaning stress in piglets. However, the effects of GABA and underlying mechanisms in weanling piglets still need further investigation. Additionally, there is limited research on how GABA affects the relative abundance of bacteria, fungi, and transcriptome changes in the ileum of weanling piglets. Therefore, this study aims to explore the impact of the dietary supplementation of GABA on bacterial and fungal composition, their metabolites, and overall intestinal function in weanling piglets.

## 2. Materials and Methods

### 2.1. Experimental Design

Weanling piglets (Duroc × Landrace × Yorkshire) at the age of 21 days with an initial body weight of 8.28 ± 0.26 kg were randomly assigned to three treatment groups with 7 replicates each. The piglets received a basal diet (CONT), a basal diet with 80 mg/kg of GABA (LGABA), or a basal diet with 120 mg/kg of GABA (HGABA). GABA (Aladdin, Shanghai, China) was incorporated into the basal diet, replacing an equivalent amount of zeolite powder. The basal diet met the nutrient requirements outlined by the National Research Council, as detailed in Supplementary Table S1. The piglets were housed individually and had ad libitum access to water and feed throughout the 28-day experimental period.

### 2.2. Growth Performance and Diarrhea Rate

Body weight and feed intake were monitored to calculate the average daily weight gain (ADG), the daily feed intake (ADFI), and the feed-to-gain ratio (F/G). Additionally, piglet diarrhea was recorded daily. The diarrhea was assessed daily following the previous study [16]. These indices were calculated using the following formula:

(1) ADG=(test final weight−test initial weight)/number of test days

ADFI = (feed intake during test − feed remaining during test)/number of test days(2)

Feed-to-Gain Ratio = average daily feed intake/average daily gain(3)

(4) $$\begin{array}{c} Diarrhea\;Ratio\;(\%)\\ \;\;\;\;\;\;=(number\;of\;piglets\;with\;diarrhea\times days\;with\;diarrhea)\\ \;\;\;\;\;\;/(total\;piglet\;number\times 28\;days)\times 100\% \end{array}$$

### 2.3. Sample Collection

At the end of the experiment, blood samples were obtained from the anterior vena cava and then centrifuged at 3000× *g* for 10 min at 4 °C; the collected serum was immediately stored at −80 °C for subsequent analysis. After that, all the piglets were euthanized using electrical stunning (250 V, 0.5 A) for 5–6 s as previously described [17]. A 2.0 cm segment of both the anterior jejunum and posterior ileum, along with 0.5 g ileal contents, were quickly collected and stored at −80 °C for further analysis. Additionally, the ileal segments were fixed in 4% paraformaldehyde for histomorphology examination.

### 2.4. Determination of Serum Biochemical Indexes

Serum biochemical indices, including aspartate aminotransferase (AST), alanine aminotransferase (ALT), and alkaline phosphatase (ALP), were detected using an automatic biochemical analyzer (Roche, Basel, Switzerland).

### 2.5. Determination of Inflammatory Cytokines and Enzyme Activities

The concentrations of inflammatory cytokines in serum and ileum, as well as the activity of lipase and trypsin in jejunum and ileum were determined using ELISA kits (BYabscience, Nanjing, China) according to the manufacturer’s instructions.

### 2.6. Intestinal Histomorphology

The ileum samples were fixed with 4% paraformaldehyde and embedded in paraffin; the section thickness was 8 μm, and the structure was observed via hematoxylin–eosin (HE) staining. SlideViewer software (version 2.8, 3DHISTECH, Budapest, Hungary) was used to measure crypt depth and villus height, and calculate the ratio of villus height to crypt depth.

### 2.7. Microbial Sequencing and Data Analysis

Microbial DNA was extracted from the ileal contents of piglets using the E.Z.N.A.^®^ Soil DNA Kit (Omega Bio-Tek, Norcross, GA, USA). The DNA quality and quantity were assessed via 2% agarose gel electrophoresis and a NanoDrop Spectrophotometer (Thermo Scientific, Waltham, MA, USA). The primer pair 341F/806R was used to amplify the bacterial 16S rRNA from the V3-V4 region, while ITS3F/ITS4R amplified the ITS2 region of the fungi. A library was constructed using the NEXTFLEX Rapid DNA-Seq Kit (Bioo Scientific, Austin, TX, USA), and sequencing was performed on the Illumina MiSeq PE300/NovaSeq PE250 platform (Illumina, Sandigego, CA, USA). FASTP software (version 0.20.0) was used for the quality control of the raw sequences, and the sequences were spliced with FLASH software (version 1.2.7, https://ccb.jhu.edu/software/FLASH/, accessed on 5 March 2024). Based on 97% similarity, OTU clustering was performed using UPARSE software (version 7.1). Microbial alpha-diversity was estimated with ACE, Chao, Shannon, Simpson, and Sobs indices, while beta-diversity was assessed using a Bray–Curtis principal coordinate analysis (PCoA) and an analysis of similarities (ANOSIM).

### 2.8. Transcriptomes

Total RNA was extracted from the ileum using TRIzol Reagent. RNA quality was assessed with a 5300 Bioanalyzer (Agilent, California, PA, USA) and quantified using the ND-2000 Spectrophotometer (NanoDrop Technologies, Wilmington, DE, USA). High-quality RNA samples were used for library construction. The Illumina NovaSeq Reagent Kit was employed to construct the sequencing library. Differentially expressed genes (DEGs) were identified based on the transcripts per million reads. Functional enrichment analyses, including a Gene Ontology (GO) analysis and the Kyoto Encyclopedia of Genes and Genomes (KEGG) pathway, were performed using Goatools and Python software (version 3.12), respectively.

### 2.9. Untargeted Metabolomics

The ileal contents were first mixed with an extraction solution (methanol: water = 4:1 (*v*:*v*)). After grinding, ultrasonic extraction, and centrifugation, the supernatant was used for liquid chromatography–mass spectrometry (LC-MS) analysis. The data were processed using Progenesis QI software (version 2.0, Waters, WWLP, Milford, MA, USA). Metabolites were identified by searching databases including Metlin (https://metlin.scripps.edu/, accessed on 10 March 2024), HMDB (http://www.hmdb.ca/, accessed on 10 March 2024), and the Majorbio Database (MJDB) from Majorbio Biotechnology Co., Ltd. (Shanghai, China). A partial least-square discriminant analysis (PLS-DA) was conducted using the R package “ropls” (Version 1.6.2). An enrichment analysis was performed with the KEGG database and the Python package (version 3.12).

### 2.10. Western Blotting

Total protein was extracted from the ileum samples, and its concentration was determined to standardize the loading amount. The proteins were denatured by heating, separated by SDS-PAGE (Thermo Scientific, Waltham, MA, USA), and transferred to a nitrocellulose membrane. The membrane was then incubated with primary antibodies against Claudin-1 (Cell Signaling, Beverly, MA, USA), Occludin (Abcam, Shanghai, China), ZO-1 (Proteintech, Rosemont, IL, USA), and GAPDH (Boster, Wuhan, China). After incubation with secondary antibodies, protein bands were detected using EZ-ECL (Biological Industries, Cromwell, CT, USA).

### 2.11. Statistical Analysis

Data were analyzed using either a one-way ANOVA followed by Duncan’s multiple comparison test or the independent samples *t*-test in SPSS Statistics 25 (SPSS Inc., Chicago, IL, USA). The values were presented as means with standard errors, and differences were considered significant when *p* < 0.05.

## 3. Results

### 3.1. Growth Performance and Diarrhea Ratio in Weanling Piglets

As shown in Table 1, based on the results from day 0–14, no significant difference was observed in BW and ADFI among the pigs in the three treatment groups. Piglets in the LGABA group exhibited a significant increase in ADG compared to the CONT group, while the HGABA group showed no significant difference. Piglets in the HGABA group exhibited a significant decrease in F/G compared to the CONT group, while the LGABA group showed no significant difference.

Based on the results from day 0–28, piglets in the LGABA group exhibited a significant increase in their final body weight (FBW) and ADG compared to the CONT group, while the HGABA group showed no significant difference. There were no significant differences in ADFI among the three groups. Both the LGABA and HGABA groups had a significant decrease in the F/G and diarrhea ratio compared to the CONT group.

### 3.2. Intestinal Inflammation in Weanling Piglets

**Figure 1:**
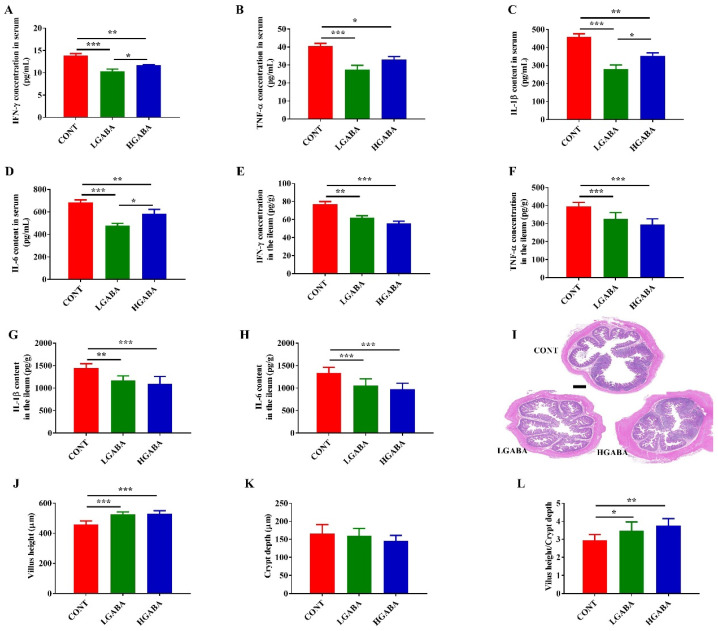
GABA supplementation alleviated intestinal inflammation in weanling piglets. Concentration of inflammatory cytokines including IFN-γ, TNF-α, IL-1β and IL-6 in serum (**A**–**D**) and ileum (**E**–**H**). (**I**) HE staining of ileum morphology (scale bar = 1.0 mm). (**J**) Villus height. (**K**) Crypt depth. (**L**) Ratio of villus height/crypt depth. Data were analyzed using one-way ANOVA followed by Duncan’s multiple comparison test (*n* = 7; mean ± SEM). * *p* < 0.05, ** *p* < 0.01, *** *p* < 0.001.

Dietary GABA significantly reduced the serum and ileal contents of pro-inflammatory cytokines, including TNF-α, IL-1β, IFN-γ, and IL-6 (Figure 1A–H). Morphological analyses showed that GABA significantly increased villus length (*p* < 0.05, Figure 1I,J). However, no significant differences in crypt depth were observed among the three groups (Figure 1K). Additionally, the villus-to-crypt ratio was higher in both the LGABA and HGABA groups (Figure 1L).

### 3.3. Microbiota Profiling in Weanling Piglets

**Figure 2:**
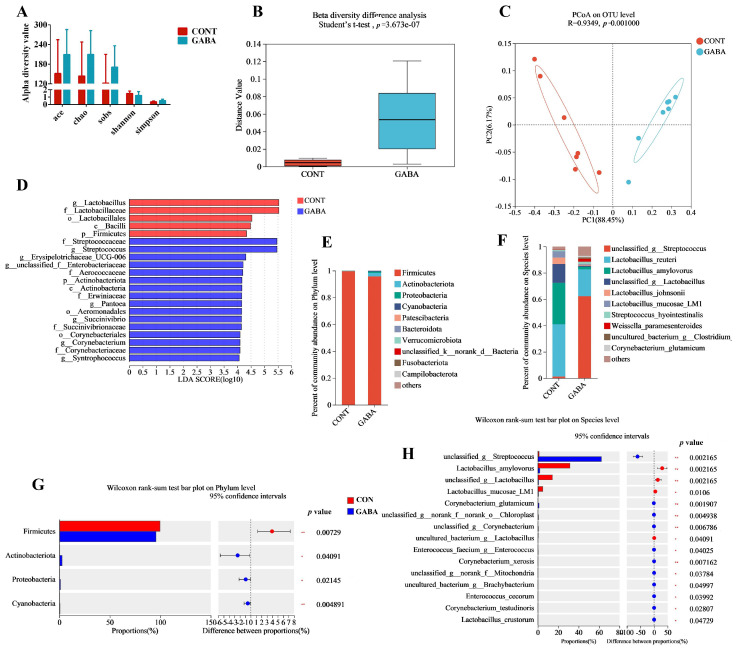
GABA supplementation-reshaped bacterial community in weanling piglets. (**A**) Alpha diversity. (**B**) Beta diversity. (**C**) PCoA on the operational taxonomic unit level. (**D**) LDA value distribution. Relative abundance of bacteria classified at phylum (**E**) and species (**F**) level. Different bacteria classified between groups at phylum (**G**) and species (**H**) level. CONT, pigs fed a basal diet. GABA, pigs fed a basal diet with 80 mg/kg of GABA supplementation. * *p* < 0.05, ** *p* < 0.01.

Since both low and high levels of GABA had similar effects on growth performance and intestinal inflammation, we focused on the low-dose GABA group, designated as the GABA group, for further analysis. We first examined microbiota composition in ileal contents via 16S rRNA sequencing. The alpha diversity showed no significant difference between the GABA and CONT groups (Figure 2A), but the beta-diversity was significantly increased with GABA supplementation (Figure 2B). PCoA revealed distinct separation in bacterial structural composition between the GABA and CONT groups (Figure 2C). The linear discriminant analysis’ (LDA) effect size (LEfSe) identified *g_Lactobacillaceae*, *f_Lactobacillus* as the dominant taxa in the CONT group, while *f_Streptococcaceae* and *g_unclassidied_f_Enterobacteriaceae* were predominant in the GABA group (Figure 2D). At the phylum level, *Firmicutes* were the most dominant, followed by *Actinobacteriota* in the GABA group (Figure 2E). At the species level, *unclassified_g_Streptococcus* was more abundant in the GABA group compared to the CONT group, and *Lactobacillus_reuteri* was the primary dominant genus. (Figure 2F). A Wilcoxon rank-sum test further confirmed that *Firmicutes* was dominant in both groups, with *Streptococcus* being more abundant and *Lactobacillus amylovorus*, and *Lactobacillus mucosae LM1* showing lower abundance in the GABA group (Figure 2G,H).

**Figure 3:**
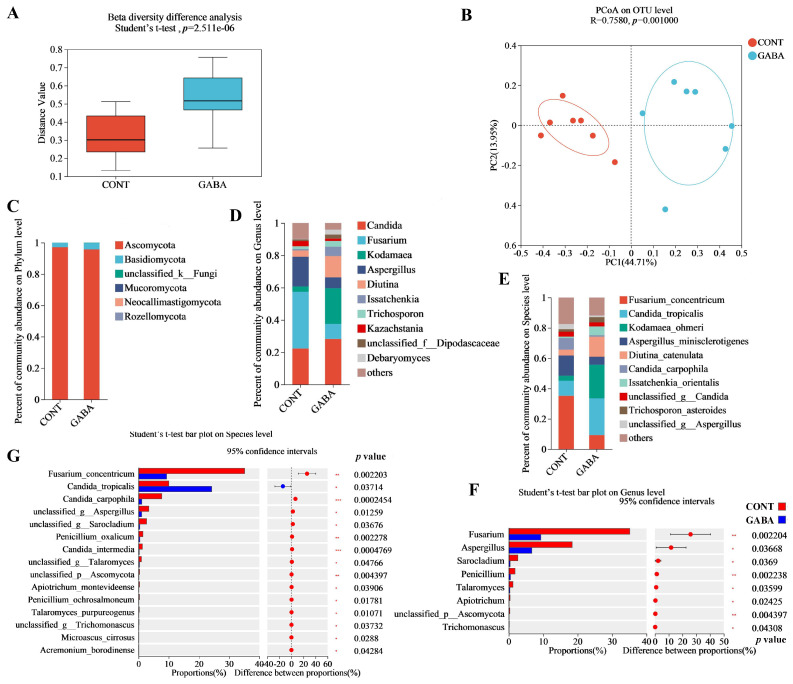
GABA supplementation-reshaped fungal community in weanling piglets. (**A**) Beta diversity. (**B**) PCoA on the operational taxonomic unit level. Relative abundance of fungi at phylum (**C**), genus (**D**) and species (**E**) level. Different fungi abundance between groups at genus (**F**) and species (**G**) level. * *p* < 0.05, ** *p* < 0.01, *** *p* < 0.001.

Similarly, fungal β-diversity showed a significant difference between the GABA and CONT groups, with distinct separation in structural composition (Figure 3A,B). Both groups were dominated by the phylum *Ascomycota* and *Basidiomycota* (Figure 3C). Compared to the CONT group, the GABA group had higher relative abundances of *Candida* and *Kodamaea*, and a lower relative abundance of *Fusarium* at the genus level (Figure 3D). At the species level, *Fusarium_concentricum* and *Candida_carpophila* were less abundant, whereas *Candida_tropicalis* and *Kodamaea_ohmeri* were more abundant in the GABA group (Figure 3E). These findings were further supported by a Student’s *t*-test (Figure 3F,G).

### 3.4. Metabolites Profiling in Weanling Piglets

**Figure 4:**
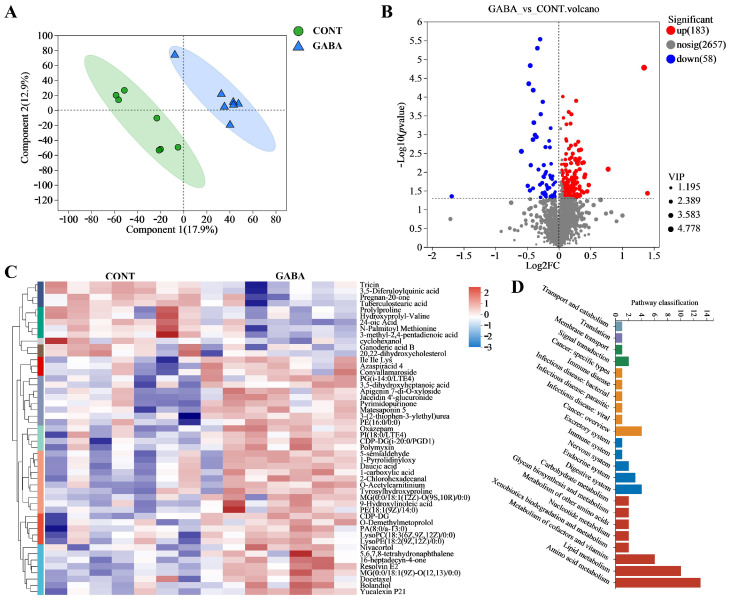
GABA supplementation-modulated intestine nutrient metabolism in weanling piglets. (**A**) PLS-DA score plot. (**B**) Volcano plot analysis of differential metabolites. (**C**) Heatmap showing the changes in metabolites. (**D**) KEGG statistical maps of differential metabolites.

To determine if the altered microbiota community influenced intestinal metabolism, a metabolomics analysis was performed. A partial least-squares discriminant analysis (PLS-DA) showed distinct differences in the metabolite composition between the GABA and CONT groups (Figure 4A). GABA treatment resulted in significant changes in 241 metabolites: 183 were upregulated, and 58 were downregulated (Figure 4B). A heatmap displayed the differential metabolites in ileal contents (Figure 4C). A KEGG enrichment analysis highlighted the top 23 enriched signaling pathways, with amino acid metabolism and lipid metabolism being the primary affected pathways (Figure 4D).

### 3.5. Transcriptional Profiling in Weanling Piglets

**Figure 5:**
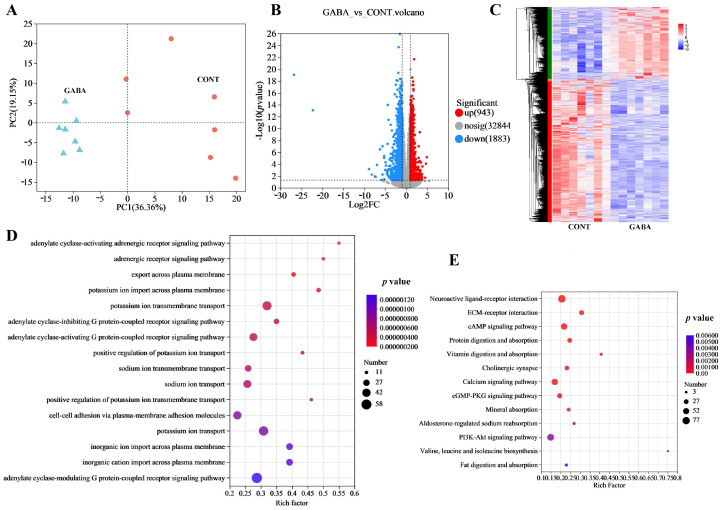
GABA supplementation-regulated transcriptional profiling in weanling piglets. (**A**) PLS-DA score plot. (**B**) Volcano plot analysis of differential expressed genes. (**C**) Heatmap showing differential expressed genes. (**D**) GO enrichment scatter plot. (**E**) KEGG enrichment scatter plot.

We then investigated the effects of GABA supplementation on ileal transcriptional profiling in weanling piglets. A principal component analysis (PCA) clearly demonstrated the significant impact of GABA on the transcriptome (Figure 5A). In the GABA group, 943 genes were upregulated, while 1883 genes were downregulated (Figure 5B). A heatmap revealed distinct differences in the expression of differential genes (Figure 5C). To identify the most affected pathways, GO and KEGG enrichment analyses were performed based on these DEGs. The GO analysis indicated that the most enriched pathways included potassium ion transmembrane transport, potassium ion transport, and the adenylate cyclase-activating G protein-coupled receptor signaling pathway (Figure 5D). Additionally, a KEGG analysis identified protein and fat absorption and digestion pathways as crucial pathways influenced by GABA supplementation (Figure 5E).

### 3.6. Digestive Enzyme Activity and Barrier Function in Weanling Piglets

**Figure 6:**
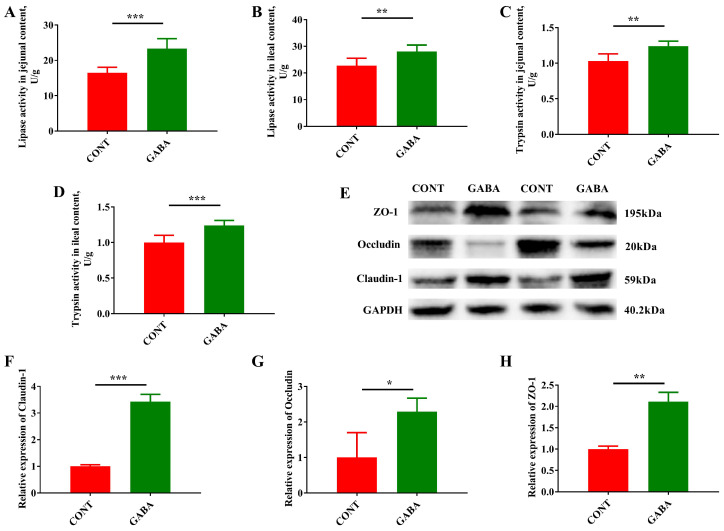
GABA supplementation-promoted digestive enzyme activity and barrier function in weanling piglets. Lipase (**A**) and trypsin (**B**) activity in jejunal contents. Lipase (**C**) and trypsin (**D**) activity in ileal contents. (**E**) Western blot measurements of tight junction protein expression (The original western blot image was provided in Supplementary Material Figure S1). Relative mRNA expression levels of Claudin-2 (**F**), Occludin (**G**) and ZO-1 (**H**) in the ileum determined by Real-time PCR. Data were analyzed by using the independent samples *t*-test (*n* = 7; mean ± SEM). * *p* < 0.05, ** *p* < 0.01, *** *p* < 0.001.

Given that intestinal metabolites and transcriptional profiling suggested improved nutrient digestion, absorption, and metabolism, we further examined the activities of specific digestive enzymes, and the expression of proteins involved in tight junction function. Compared to the CONT group, the activity of lipase and trypsin in the jejunal and ileal contents was significantly higher in the GABA group (Figure 6A–D). Additionally, the expression of Claudin-1, Claudin-2, and ZO-1 proteins in the ileum was notably increased following GABA supplementation (Figure 6E–H).

## 4. Discussion

GABA has garnered attention for its roles in alleviating oxidative stress and modulating intestinal microbiota [11,12,18]. Recent studies have highlighted its benefits in enhancing intestinal immunity and increasing bacterial diversity in weanling piglets [11,19]. Our study further demonstrated that dietary GABA improves body weight gain and decreases the diarrhea ratio in weanling piglets. Additionally, GABA supplementation decreased pro-inflammatory cytokine levels and improved intestinal morphology, indicating a reduction in intestinal inflammation. Notably, a high dose of dietary GABA did not demonstrate better effects on growth performance and inflammation compared to a low dose of GABA. Recent evidence indicated that the stimulatory impact of higher GABA doses on the secretion of cholecystokinin, a hormone known to modulate satiety, thereby led to a reduction in the appetitive behavior of the piglets [20,21]. Therefore, we focused on the piglets who were fed a low dose of GABA for the subsequent analyses. We observed increased beta-diversity in both the bacteria and the fungi, with several previously low-abundance species becoming dominant, suggesting that 80 mg/kg GABA impacts microbial composition. Metabolomics revealed changes in lipid and amino acid metabolism, while a transcriptome analysis identified enrichment in ion transmembrane transport and adenylate cyclase signaling pathways, and protein and fat absorption and digestion pathways. These findings suggested that 80 mg/kg dietary GABA enhances nutrient absorption and metabolism.

Weaning stress poses a significant threat to the swine industry, often leading to impaired intestinal morphology, weakened barrier function, dysbiosis, increased diarrhea and growth retardation [3]. Maintaining cytokine levels is essential for intestinal health; elevated levels of pro-inflammatory cytokines, such as TNF-α and IL-1β, are commonly observed in weanling piglets [22]. These elevated cytokines contribute to inflammation and autoimmunity [23], further impairing intestinal function. Previous studies have shown that GABA can reduce the secretion of inflammatory cytokines by interacting with receptors on immune cells [24,25]. Mechanistically, the inhibitory effect of GABA on these cytokines may result from its direct action on functional GABA receptors in antigen-presenting cells, leading to a reduction in MAPK signaling [25]. Additionally, GABA may regulate the responses of pro-inflammatory macrophages, thereby contributing to the management of cytokine levels and overall inflammatory status [26]. Additionally, improved intestinal morphology further supports the role of GABA in mitigating inflammation and protecting the intestinal barrier against pro-inflammatory substances like LPS [27]. Tight junctions, composed of transmembrane proteins and intracellular plaque proteins, including Claudin, Occludin, and ZO, are crucial for the maintenance of the intestinal barrier integrity [28]. They serve as a physical barrier against inflammatory cytokines and pathogens, and help protect the underlying tissues [29,30]. Additional GABA can enhance intestinal integrity by increasing the relative abundance of tight junction proteins [14]. This boost in protein levels strengthens the barrier function, protects against permeability issues, and maintains overall gut health; thus, dietary GABA ameliorate the intestinal impairments caused by weaning in piglets.

Microbiota play a crucial role in the intestine, influencing various physiological processes, including inflammation and diarrhea [31,32,33,34]. A healthy microbiota community protects against dysbiosis-related diseases. High diversity within the intestinal microbiota is crucial for resisting adverse conditions and maintaining a stable microbial ecosystem [35,36]. When exposed to external perturbations such as bacterial infections and environmental challenges, a diverse microbiota is more effective at maintaining microbial equilibrium, which supports host health. Evidence indicates that as species diversity increases, the ability of a microbial community to resist pathogen colonization also significantly improves [37]. Furthermore, the metabolites derived from the microbiota play a crucial role in the crosstalk between the microbiota and the intestine, as well as in the regulation of inflammation [38]. The intricate diversity within the gut microbiome facilitates the synthesis of a wide range of beneficial metabolites. Numerous studies have indicated that GABA supplementation can promote the growth of specific microbiota, suggesting that GABA acts as a key growth factor in regulating microbial abundance [39]. In our study, GABA supplementation significantly increased the β-diversity of ileal bacteria and fungi, leading to the emergence of many previously low-abundance microorganisms. This increased microbiota diversity supported intestinal health and enhanced the ability to resist negative disruptions, which may explain the observed reduction in the pro-inflammatory cytokines following GABA supplementation. Additionally, GABA may exert a protective influence in the intestinal tract by modulating the dynamics of low-abundance microbial communities. Our investigation revealed an increase in the prevalence of *Enterococcus faecium*, a member of the *Enterococcus* genus, following GABA supplementation. *Enterococcus faecium* showed promise for probiotic use in the swine industry, as it has the potential to enhance IgA responses, which are crucial for maintaining intestinal mucosal immunity and defending against pathological infections [40,41,42]. In addition, GABA supplementation seemed to protect the intestine against pathogenic incursions by decreasing the incidence of pathogenic fungi, such as *Fusarium concentricum* and *Candida carpophila* [43,44]. Thus, modulating the diversity and composition of intestinal bacterial and fungal populations may be a key mechanism through which GABA promotes gastrointestinal well-being.

Elevated levels of digestive enzymes reflect enhanced capabilities for digestion, absorption, and nutrient metabolism, contributing to better growth performance [45]. In this study, GABA supplementation enriched amino acid and fatty acid metabolism pathways and increased the relative expression of lipase and trypsin, indicating that GABA may protect the intestine by modulating nutrient metabolism. The underlying mechanism by which GABA affects nutrient metabolism could be related to changes in intestinal microbiota composition, as microbiota colonization impacts digestive enzyme activity [46] and influences intestinal permeability [47]. Additionally, improvements in intestinal morphology and nutrient absorption due to GABA supplementation may further increase lipase activity, enhancing overall nutrient metabolism [27].

A transcriptome analysis revealed that GABA supplementation enriched ion transport and G-protein coupled receptor (GPCR) signaling pathways in the intestine. Ion transport proteins are crucial for managing inflammation-related diarrhea and maintaining intestinal mucosal integrity. A decrease in these proteins can impair resistance to pathological factors [48]. Thus, the enrichment of ion transport pathways suggests improved resistance to disturbances in weanling piglets following GABA supplementation. The intestine contains several receptors for GABA, including ionotropic GABAA and GABAC receptors, as well as metabotropic GABAB receptors, all of which belong to the G protein-coupled receptor (GPCR) family [8]. GPCRs constitute the most diverse group of cell membrane receptors and play a significant role in inflammatory bowel diseases [49]. For example, GPCR120 acts as a receptor for omega-3 fatty acids, helping to prevent macrophage-induced tissue inflammation [50]. The enrichment of GPCR-signaling pathways supports a favorable intestinal environment [51], as GPCRs also regulate metabolism of amino acids, fatty acids, and bile acids [52]. Additionally, metabotropic GABA receptors, which couple with Gi/o class G proteins, play roles in slow and prolonged signaling [53]. Thus, GABA may modulate intestinal function by targeting GPCRs and other signaling pathways, contributing to a healthier intestinal environment.

## 5. Conclusions

In conclusion, our study indicates that dietary 80 mg/kg GABA enhanced growth performance, reduced diarrhea, and improved both intestinal morphology and inflammatory response in weanling piglets. Multi-omics analyses revealed that 80 mg/kg GABA supplementation positively influenced nutrient digestion, absorption, and metabolism, which likely underpinned its beneficial effects. These findings are further supported by the increased activity of digestive enzymes and the higher expression of tight junction proteins. Our research enhances the understanding of the mechanisms of GABA in alleviating weaning stress and further confirms that GABA has the potential to mitigate the challenges associated with weaning. However, we still know very little about the impact of GABA on fungal flora and the mechanisms by which fungi affect the intestinal health of weanling piglets. In addition, the underlying mechanisms regarding the effect of GABA on nutrient absorption and ion transport pathways require further exploration.

## Figures and Tables

**Table 1:** Growth performance in weanling piglets.

	CONT	LGABA	HGABA
IBW, kg	8.77 ± 0.54	8.64 ± 0.47	8.63 ± 0.38
Day 0–14			
BW, kg	12.75 ± 0.86	13.79 ± 0.64	13.45 ± 0.41
ADG, g	282 ± 28 ^a^	369 ± 21 ^b^	344 ± 26 ^ab^
ADFI, g	523 ± 49	557 ± 32	470 ± 49
F/G	1.94 ± 0.27 ^a^	1.52 ± 0.06 ^ab^	1.36 ± 0.08 ^b^
Day 0–28			
BW, kg	17.71 ± 1.08 ^a^	21.17 ± 1.18 ^b^	19.66 ± 0.91 ^ab^
ADG, g	319 ± 27 ^a^	448 ± 32 ^b^	394 ± 36 ^ab^
ADFI, g	630 ± 47	746 ± 45	659 ± 70
F/G	2.06 ± 0.25 ^a^	1.69 ± 0.11 ^b^	1.68 ± 0.08 ^b^
Diarrhea, %	9.34 ± 1.07 ^a^	2.49 ± 0.34 ^b^	3.23 ± 0.56 ^b^

Data were analyzed using one-way ANOVA followed by Duncan’s multiple comparison test (*n* = 7; mean ± SEM). ^a,b^ within a row, means with different superscript letters are different (*p* < 0.05). CONT, pigs fed a basal diet; LGABA, pigs fed a basal diet with 80 mg/kg GABA. HGABA, pigs fed a basal diet with 120 mg/kg GABA.

## Data Availability

The 16S rRNA gene sequence data and RNA-Seq data have been deposited in the NCBI Bioproject database (https://www.ncbi.nlm.nih.gov/bioproject/, accessed on 30 March 2024), registration number for PRJNA1087290 and PRJNA1083413, respectively.

## Supplementary Materials

The following supporting information can be downloaded at: https://www.mdpi.com/article/10.3390/ani14223177/s1, Table S1: Basal diet composition and nutrient level, Figure S1: Original image of western blotting.
