# Supplementary material for: Multi-Omics Reveal the Improvements of Nutrient Digestion, Absorption, and Metabolism and Intestinal Function via GABA Supplementation in Weanling Piglets
Source: Animals (Basel). 2024 Nov 6;14(22):3177. doi: 10.3390/ani14223177 (PMC11591204; doi:10.3390/ani14223177)
Supplement: Supplementary file 1 [file animals-14-03177-s001.zip › Figure S1.pdf]

Figure S1 Original image of western blotting

A

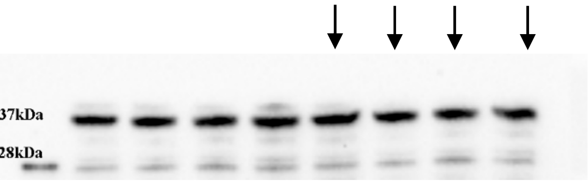

B

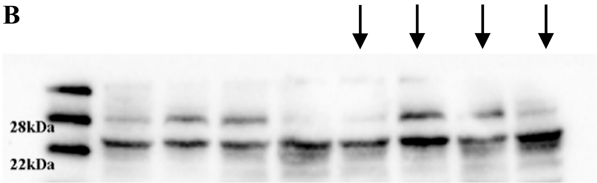

C

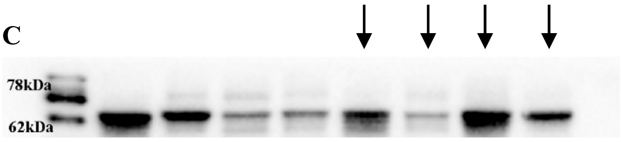

D

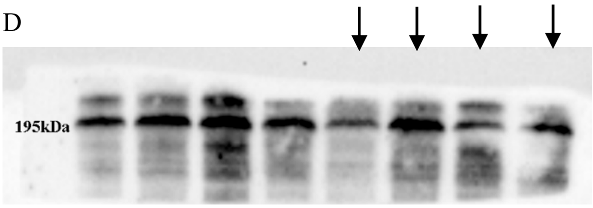

A, GAPDH; B, Claudin-1; C, Occludin; D, ZO-1; The lane indicated by the arrow is applied.
