# Supplementary material for: Multi-Omics Reveal the Improvements of Nutrient Digestion, Absorption, and Metabolism and Intestinal Function via GABA Supplementation in Weanling Piglets
Source: Animals (Basel). 2024 Nov 6;14(22):3177. doi: 10.3390/ani14223177 (PMC11591204; doi:10.3390/ani14223177)
Supplement: Supplementary file 1 [file animals-14-03177-s001.zip › supplementary materials.pdf]

Supplementary Table 1 Basal diet composition and nutrient level

Item	%	Calculated nutrient level	
Corn	52.00	Calculated nutrient level	
Extruded soybean	6.00	DE, MC/kg	3.52
Broken rice	11.00	CP, %	17.49
Soybean meal[1]	16.00	Ca, %	0.81
Whey powder	5.00	Total P, %	0.63
Fish meal	3.00	P, %	0.41
Coconut oil	1.00	Lysine, %	1.21
Soybean oil	1.00	Methionine, %	0.37
Limestone	0.80	Threonine, %	0.73
calcium hydrogen phosphate	1.30	Tryptophan, %	0.21
NaCl	0.30	Analyzed nutrient level	
L-lysine hydrochloride	0.50	DE, MC/kg	3.34
DL-methionine	0.10	CP, %	17.71
L-threonine	0.20		
L-tryptophan	0.06		
Vitamin[2]	0.03		
Mineral[3]	0.10		
Zeolite powder	1.61		
Total	100		

CP = crude protein; DE = digestible energy. [1] Soybean meal contains 43% protein. [2] Provided for per kilogram of diet: vitamin A, 32.5 million IU; vitamin B_1, 10 g; vitamin B_2, 25 g; vitamin B_6, 15 g; vitamin B_{12}, 100 mg; vitamin D_3, 10 million IU; vitamin E, 80 g; vitamin K_3, 10 g; nicotinamide, 120 g; D-pantothenic acid, 70 g; folic acid, 5 g; D-biotin, 500 mg. [3] Provided for per kilogram of diet: $FeSO_4 \cdot H_2O$, 348.97 g; $CuSO_4 \cdot 5H_2O$, 48.83 g; $ZnSO_4 \cdot H_2O$, 193.21 g; $MnSO_4 \cdot H_2O$, 84.51 g; $Ca(IO_3)_2 \cdot H_2O$, 0.768 g; $Na_2SeO_3 \cdot 5H_2O$, 1.18 g; Fe, 115 g; Cu, 12.5 g; Zn, 70 g; Mn, 27.5 g; I, 0.5 g; Se, 0.35 g.
